# Novel mechanistic study of HDAC6 regulation of rheumatoid arthritis via CMA: exploring potential therapeutic targets

**DOI:** 10.3389/fphar.2024.1383663

**Published:** 2024-03-21

**Authors:** Duoduo Lin, Weipeng Lai, Ningning Zheng, Hongbin Luo, Xiaole Chen, Wenzhong Que, Nanwen Zhang

**Affiliations:** ^1^ The School of Pharmacy, Fujian Medical University, Fuzhou, Fujian, China; ^2^ Putian Lanhai Nuclear Medicine Research Center, Putian, Fujian, China; ^3^ Key Laboratory of Natural Medicine Pharmacology in Fujian Province, Fuzhou, Fujian, China; ^4^ Department of Sports Medicine, The First Affiliated Hospital of Fujian Medical University, Fuzhou, Fujian, China; ^5^ Department of Rheumatology, Fuzhou No. 1 Hospital Affiliated with Fujian Medical University, Fuzhou, Fujian, China

**Keywords:** rheumatoid arthritis, histone deacetylase 6, heat shock protein, molecular chaperone autophagy, therapeutic target

## Abstract

**Objective:**

Rheumatoid arthritis (RA) is a systemic autoimmune disease. Its pathogenesis has not yet been clarified, so it is urgent to explore therapeutic targets. Here, we clarified the role of HDAC6 in the mechanism of action of RA through mediating chaperone-mediated autophagy (CMA) to provide a clinical treatment of RA.

**Methods:**

We used rheumatoid arthritis fibroblast-like synoviocytes (RA-FLS) and collagen-induced arthritis mice (CIA mice) as models of RA and pharmacological inhibitors as well as genetic interference with adeno-associated viruses to reduce the expression of HDAC6. We explored the influence of CAY10603 on RA-FLS proliferation and inflammation, as well as the expression of proteins related to the CMA signaling pathway. CIA model was constructed using DBA/1J mice. Arthritis symptoms in CIA mice were evaluated, and the expression and localization of CMA-related proteins in mouse ankle joints were examined.

**Results:**

CAY10603 inhibited proliferation as well as the level of the molecular chaperone autophagy in RA-FLS. HDAC6 shRNA significantly reduced the clinical signs of arthritis in CIA mice, as did the expression of HDAC6 in the serum and ankle synovial tissues of CIA mice. Finally, it significantly inhibited the level of Hsc70 and LAMP-2A, which are involved in the CMA signaling pathway, in ankle joint tissues.

**Conclusion:**

Downregulation of HDAC6 may inhibit CMA and thereby ameliorate RA.

## Introduction

Rheumatoid arthritis (RA) a chronic autoimmune condition, primarily affects peripheral joints, leading to joint deformities. And it is recognized pathologically by continuous synovitis, vascular opacification and skeleton erosion (Davis et al., 2020). Epidemiological surveys have shown that the global prevalence of RA is 0.24% (Almutairi KB. et al., 2021; Almutairi K. et al., 2021), but its pathogenesis has not been elucidated; however, abnormal proliferation of synovial tissue is considered to play a critical part in the development of RA (Fraenkel et al., 2021).

Histone deacetylase-6 (HDAC6), belonging to the class II histone deacetylases, possesses both a ZnF ubiquitin-binding structural domain and a dynamin-binding structural domain. HDAC6 combines with both ubiquitinated and misfolded proteins along microtubule orbits pericentromeric structures and promotes aggregate formation, which subsequently activate autophagy to remove cytotoxic protein aggregates (Kaur et al., 2022). HDAC6 exerts its biological effects mainly as a deacetylase, and common substrates include α-microtubulin (Shi et al., 2022), cortactin (Luthold et al., 2020)and Hsp90 (Du et al., 2021). Downregulation of HDAC6 inhibits the inflammatory reaction and invasion of FLS in RA (Bae et al., 2021; Park et al., 2021). The above studies indicates that HDAC6 may be a potential objective for inhibiting RA.

There are three main types of autophagy in eukaryotic cells: macroautophagy (hereinafter referred to as autophagy), microautophagy and molecular chaperone-mediated autophagy (CMA). CMA selectively delivers proteins to lysosomes for degradation. The process involves three stages: substrate identification, substrate deployment and transportation, and substrate degradation. During substrate recognition, heat shock protein 70 (Hsc70) discriminates the KFERQ motif in substrate proteins (Su et al., 2016) and other cochaperones to creat a chaperone-substrate complex. Then, at the stage of substrate unfolding and translocation, the chaperone-substrate complex binds to lysosome-associated membrane protein type 2A (LAMP-2A), the substrate unfolds with the help of the chaperone, and the unfolded substrate is transferred to the lysosome by polymerized LAMP-2A (Aoyagi and Archer, 2005). Finally, at the stage of substrate degradation, lysosomal hydrolases breakdown the substrate into amino acids, which can be reused by the cell, and LAMP-2A dissociates from the chaperone complex and returns to the monomeric state (Du et al., 2021). CMAs are critical for protein function, from facilitating folding, translocation and translocation through functional maturation to removing misfolded and aggregated material through the UPS, autophagy or lysosomal pathways. Dysfunction of CMA has proinflammatory effects. It was found that defective CMA promotes inflammation (Qiao et al., 2021).

Here, we found that synovial tissue-expressed HDAC6 was positively associated with RA both in a mouse model and in human participants. HDAC6 loss regulates CMA to ameliorate RA.

## Materials and methods

### RNA seq and data analysis

A data matrix was obtained from Gene Expression Omnibus (GSE206848, RA two samples vs. Normal seven samples), the Prob IDs were converted to gene symbols, and a differential analysis was performed using limma (parameters: screen Foldchange>1.5, *p* ≤ 0.05 as the screening threshold) to screen for markedly differentially expressed genes and then create a graph. The top 100 and HDAC6 genes were upregulated according to the constructed PPI network, after which the PPI relationships were screened with a score≥0.4 as the threshold.

### Mouse model and cell

Sixteen healthy male DBA1/J mice (6–8 weeks; 18–20 g) were used in this study (Jiangsu Jicui Yaokang Biotechnology Co., Ltd.). The room temperature of the experimental animal housing was 20°C–26°C, relative humidity was 40%–70%, and the day/night cycle was 12 h. All mice were fed and watered freely, bedding was changed daily, and feed was provided by the Experimental Animal Center. All experimental protocols for mice complied with the regulations of the International Committee for the Protection of Animals and the Local Commission for the Consumption and Protection of Laboratory Animals, No. (No): 2023-Y-0077. Mice were grouped into four groups: the control group, model group, EGFP-NC group and HDAC6 shRNA group.

RA-FLS were incubated at 37°C in an incubator with 5% CO_2_. RA-FLS (Hunan Fenghui Biotechnology Co., Ltd.) was cultured in DMEM (Gibco, USA) containing 10% fetal bovine serum (FBS, PAN). The cells were divided into three groups: the RA-FLS group, CAY10603 intervention group, and tamoxifen intervention group.

### CCK8 assay

RA-FLS were placed in a 96-well plate (5×10^3^ cells/well) and cultured overnight. In the drug administration group, 10 μM CAY10603 was added, six replicate wells were established for each group, and then incubated at 37°C for 24 h. Following the addition of CCK8 solution (Beyotime Biotechnology #C0037) to each well, the cells were incubated at 37°C for 1 h. Subsequently, their OD values were measured at 450 nm using an enzyme marker.

### EdU assay

RA-FLS (5×10^3^ cells/well) were placed in 96-well plates, cultured and transfected for 2 h. We used the EdU-448 Cell Proliferation Kit (Beyotime Biotechnology #C0071S), and an aliquot of EdU working solution (20 μM) pre-warmed at 37°C was added to a 6-well plate and diluted to 10 μM. And stained for nuclear with Hoechst 33342, and imaged and analysed for EdU with Thermo Scientific Cellomics ArrayScan VTI HCS (Thermo Fisher Scientific, USA) for EdU imaging and analysis. The ratio of EdU-positive cells in green to RA-FLS-positive cells in blue was used to calculate the percentage of EdU binding.

### ELISA

Peripheral blood from the orbital venous sinus of mice as well as supernatants of RA-FLS cells were taken, and the levels of TNF-α (human: SMK0122B), IL-6 (human: SMK0049B), and HDAC6 (mouse: SMK6102A) in serum or supernatants were analyzed by ELISA based on the protocols (Jiangsu Sumida Biological Co., Ltd.).

### Western blot analysis

Wash the cells slowly with PBS (DINGGUO, BF-0011) and repeat 3 times, then lysed on ice using RIPA Lysate Agent (Beyotime Biotechnology #P0013B) for 30 min. The protein concentration of the samples was determined by BCA protein assay kit (GLPBIO #GK10009). Proteins were separated by 12% dodecylsulfonyl polyacrylamide gel electrophoresis (SDS‒PAGE), and the separated proteins were shifted to polyvinylidene fluoride (PVDF) membranes (FFP28, Beyotime Biotechnology Ltd.). The PVDF membranes were placed on a shaking bed in a sealing solution for 1.5 h. Then, rabbit HDAC6 (Cell Signaling Technology, D21B10), LAMP2A (Abcam, ab125068), Hsc70 (Abcam, ab51052), LC3B (Abcam, ab192890), SQSTM1/p62 (Abcam, ab109012), β-actin (Cell Signaling Technology, 4967S), and β-Tubulin (Proteintech, 2146S) were identified using primary antibody overnight at 4°C in the refrigerator. Incubate with goat anti-rabbit IgG (Abcam, ab6721) secondary antibody for 1.5 h, then washed with 0.1% TBST. At the end of three washes, observation was performed with a chemiluminescence visualiser (Shanghai Qinxiang Scientific Instrument Co., Ltd.).

### Immunocytochemistry (ICC)

RA-FLS were treated with 10 μM CAY10603 (Abmole, M38888-01) for 24 h, secured with 4% paraformaldehyde (Servicebio, G1101), penetrated with 0.1% Triton-X-100 (Beyotime Biotechnology, ST1722), and blotted with 2% BSA (Servicebio, G5001). LAMP2A (Proteintech, 66301-1-Ig1:200) and Hsc70 (Abcam, ab51052, 1:200) antibodies were applied to the treated cells, which were subsequently incubated overnight at 4°C. Alexa Fluor^®^ 488 (Affinity, S0018, green) and Alexa Fluor^®^ 594 (Affinity, S0007, red) secondary antibodies (1:200) were added, and the samples were incubated for 1.5–2 h before being and imaged using a Leica confocal imaging system.

### Introduction of CIA

Male DBA1/J mice were randomly grouped. In the CIA group, an emulsion of bovine type II collagen (Chondrex #2022) and complete Freund’s adjuvant (CFA, Chondrex #7008) at a terminal viscosity of 0.5 mg/mL was injected subcutaneously into the caudal root on day 0, and an emulsion of collagen and incomplete Freund’s adjuvant (IFA, Chondrex #7002) was injected on day 21. DBA/1J mice gradually developed signs of arthritis 28–35 days after first immunization. Then mice were randomly grouped into four groups based on CIA swelling and scoring (Christman et al., 2023): the control group, CIA group, EGFP NC group (Hanbio, China) and HDAC6 shRNA group (Hanbio, China). Ten microliters of EGFP NC and 10 μL of HDAC6 shRNA were administered every 6 days for a total of three injections. During the period of administration, we injected pbs into normal and CIA mice. After the first immunization, the mice were scored every 3 days and their hind paws were photographed. The arthritis index scores were as follows: 0 represents normal, one represents measurable arthritis as well as erythema, two represents significant swelling and redness, three represents severe swelling of the joints and fingers as well as redness, and four represents the most severe swelling and deformity with ankylosis. The cumulative value of the four paw disease scores is the total arthritis score, up to a maximum of 16 points.

Score for number of swollen arthritic feet and paws: one ankle and five toe joints of each foot were observed for swelling and the swelling score was recorded up to a maximum of 24 points per mouse (Wang et al., 2021). Paw thickness was analyzed by measuring the average thickness of the hind paw of each mouse using a digital micrometer (Nass et al., 2021).

### Histopathological examination

At the end of drug administration, mice were anaesthetized with pentobarbital. The right hind limbs were fixed with 4% paraformaldehyde for 1 day. Dehydrate it using alcohol and embed it in paraffin. Tissue samples of 2.0 cm × 2.0 cm × 0.3 cm were sliced into 3 μM-sized slices using a Leica ultrathin microtome (Leica, Germany). The tissue samples were then stained with H&E, toluidine blue, and safrole O.

Three independent observers rated the seriousness of inflammation and chondral lesions. Below are scoring criteria.: 1) synovial inflammation, 0 indicates normal, one indicates a slight diffuse inflammatory infiltrate, two indicates medium inflammatory infiltrate, and 3indicates severe inflammation with abscess. 2) Cartilage lesions and bone erosion: 0 indicates normal, 1indicates gentle loss of staining, without marked chondrocyte loss, two indicates medium with focal gentle chondrocyte loss, and three indicates serious diffuse staining loss and chondrocyte loss (Li et al., 2020).

### Immunohistochemistry staining

Paraffin sections were made after deparaffinizing in water and antigenically repaired using ready-to-use pepsin repair solution. Endogenous peroxidase was blocked with 3% hydrogen peroxide. Sections were subjected to 3% bovine serum albumin (BSA) for 1.5–2 h, then anti-HDAC6 antibody was added dropwise and incubated overnight at 4°C. The slices were then cleaned with phosphate-buffered saline (PBS) and the appropriate secondary antibody was incubated with HRP for 50 min at room temperature. DAB development was then performed and the nuclei were counterstained by adding hematoxylin-eosin. The following wavelengths were used: DAPI, ultraviolet excitation wavelength 330–380 nm; emission wavelength, 420 nm; blue light; FITC, excitation wavelength, 465–495 nm; emission wavelength, 515–555 nm; green light; and FITC, excitation wavelength, 590 nm; emission wavelength, 510–561 nm; red light.

### Statistical analysis

The results are presented as the mean ± standard deviation and using two-tailed Student’s t-test and one-way ANOVA. No data were excluded from the analyses. Statistical analysis was performed using GraphPad Prism 8.1 software, and *p* < 0.05 stands for statistical significance.

## Results

### Synovial tissue-expressed HDAC6 enhances autoimmune arthritis

**FIGURE 1:**
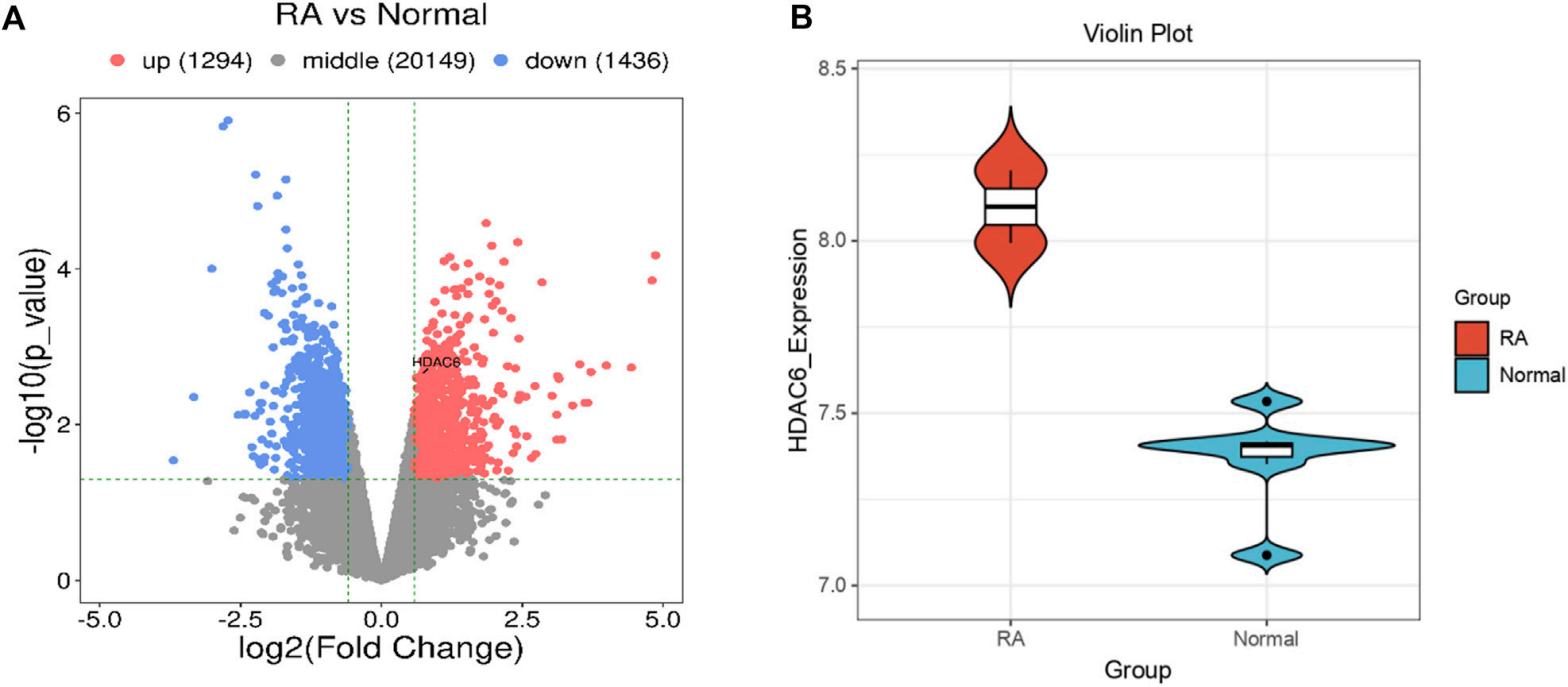
HDAC6 is highly expressed in the synovium of RA patients. **(A, B)** Comparison of HDAC6 transcript levels in the synovium between healthy controls (HCs) and participants with RA from publicly available transcriptome data **(A)** = volcano plot, **(B)** = violin plot).

To address whether and how HDAC6 is associated with RA, we analyzed publicly available transcriptome data from the synovium of participants with RA. HDAC6 expression in the synovium of participants with RA was greater than that in the synovium of healthy controls (Figures 1A, B).

### HDAC6 loss inhibits the proliferation of RA-FLS

**FIGURE 2:**
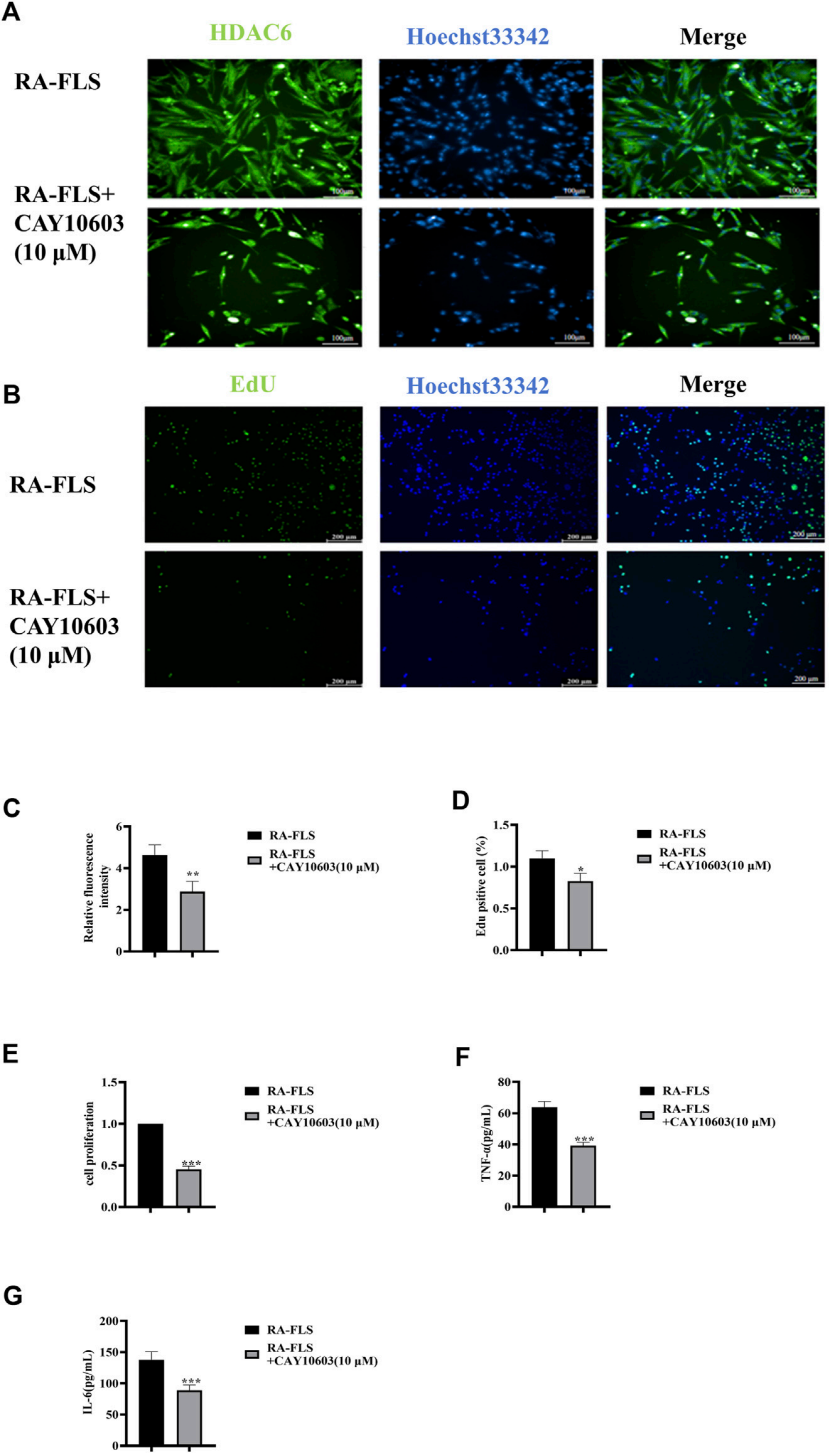
CAY10603 inhibits proliferation and inflammatory factor secretion in RA-FLS. **(A)** Representative image (20×) of the ability of the EdU assay to detect the proliferation of RA-FLS after 24 h of drug intervention (n = 6 per group). Blue fluorescence indicates total cells, and green fluorescence indicates proliferating cells. **(B)** Representative image (10×) of the migration of RA-FLS detected via an immunofluorescence assay after 24 h of drug intervention (n = 6 per group). **(C)** ImageJ analysis of the number of proliferating cells determined by EdU staining. **(D)** ImageJ analysis of the number of proliferating cells determined by the CCK8 assay. **(E)** Statistical analysis of the immunofluorescence results. **(E-F)** The expression levels of TNF-α and IL-6 were determined via ELISA. The results are expressed as the mean ± SD. All the experiments were repeated three times. ^*^ *p* < 0.05, ^**^ *p* < 0.01, ^***^ *p* < 0.001 vs. the RA-FLS group.

CAY10603 (BML-281) is a highly efficiency and specific inhibitor of HDAC6. HDAC6 is cytoplasmically localized (Jo et al., 2022), and to observe its intracellular localization, immunofluorescence was used to observe HDAC6 in RA-FLS (Figures 2A, C). The results suggested that CAY10603 was able to effectively restrain the level of HDAC6 in RA-FLS (*p* < 0.01). To verify whether CAY10603 has an inhibitory effect on the proliferation of RA-FLS, we employed CCK8 and EdU assays to evaluate the proliferation of RA-FLS, and the results showed (Figure 2B, D-E) that 10 μM CAY10603 could efficiently restrain the proliferative effect of RA-FLS compared with that of the RA-FLS group (*p* < 0.05, *p* < 0.01, *p* < 0.001). The cell supernatants were collected after the drug administration, and changes in inflammatory factor levels were detected via ELISA. As shown in the graphs (Figures 2F, G), the secretion levels of TNF-α and IL-6 in RA-FLS were significantly diminished by CAY10603 (*p* < 0.001), suggesting its effective inhibition of inflammatory progression.

### HDAC6 loss inhibits the autophagy of molecular chaperones in RA-FLS

**FIGURE 3:**
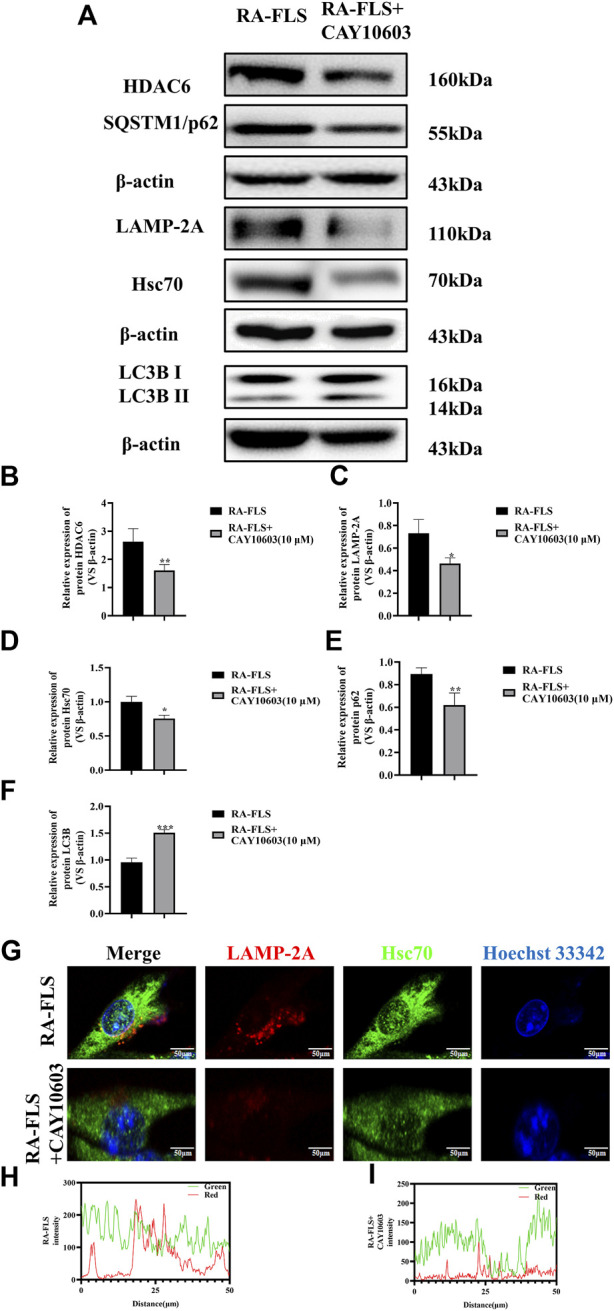
Effect of CAY10603 on the chaperone molecular autophagy in RA-FLS. **(A–F)** Western blotting was used to detect the expression of autophagy pathway-related proteins in RA-FLS. **(G–I)**: Confocal detection of Hsc70 and LAMP-2A expression in RA-FLS. ^*^ *p* < 0.05, ^**^ *p* < 0.01, ^***^ *p* < 0.001 vs. the RA-FLS group.

To verify whether CAY10603 affects autophagy in RA-FLS, the activation of the HDAC6, Hsc70, and LAMP-2A signaling pathways in RA-FLS was assessed by Western blotting (Figures 3A–D). The results revealed that the levels of HDAC6, Hsc70, and LAMP-2A were greatly downregulated after the administration of 10 μM CAY10603, indicating that CAY10603 inhibited the activation of the CMA pathway (*p* < 0.05, *p* < 0.01). Moreover, CAY10603 upregulated LC3B expression and downregulated p62 expression (*p* < 0.01, *p* < 0.001), suggesting that the degree of autophagy was decreased. To assess the colocalized expression of Hsc70 and LAMP-2A in RA-FLS cells, they were observed using the confocal method, and it was found (Figures 3G–I) that CAY10603 downregulated the expression of Hsc70 and LAMP-2A, and at the same time, inhibited the degree of colocalization of the two. HDAC6 loss effectively improved the clinical signs and ameliorated inflammation and bone damage in CIA mice.

**FIGURE 4:**
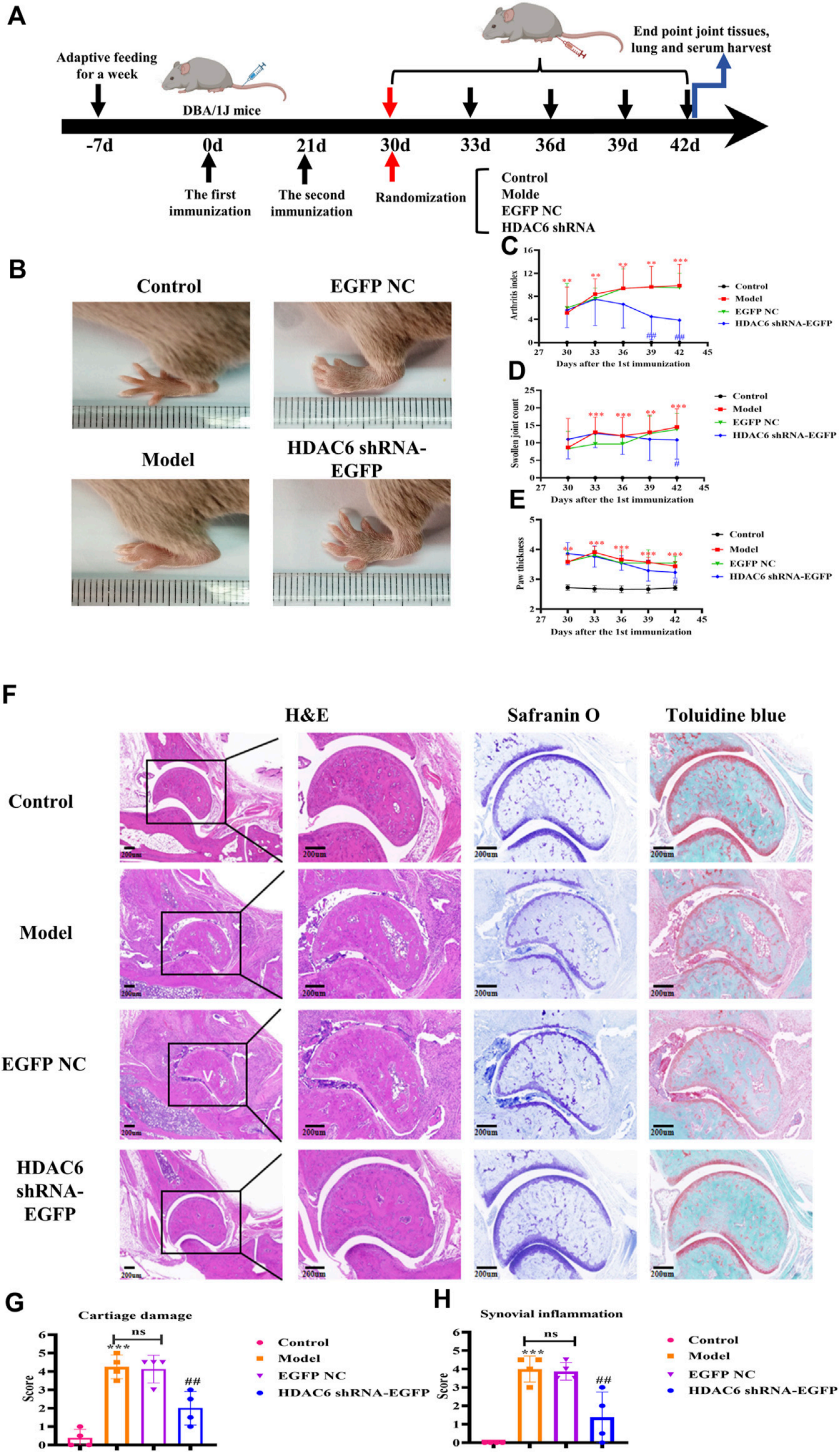
Effect of HDAC6 shRNA on clinical symptoms of arthritis in CIA mice. **(A)** Schematic of CIA mouse model induction and HDAC6 shRNA intervention. **(B)** Representative images of hind paws from different groups of CIA mice on the 42nd day. **(C)** Rthritis indices of CIA mice. **(D)** Swelling index. **(E)** Claw thickness measurement. ^**^ *p* < 0.01, ^***^ *p* < 0.001 vs. the control group; ^#^ *p* < 0.05, ^##^ *p* < 0.01 vs. the CIA group. **(F)** Representative H&E, toluidine blue and safranine histological images of hind paws obtained from the HDAC6 shRNA group on the 42nd day. **(G)** The histology scores of synovial inflammation. **(H)** Histological scoring of bone and cartilage injuries.

To verify the therapeutic treatment of HDAC6 shRNA on RA, we constructed a CIA mouse model broadly employed to elucidate the mechanism of action of RA and to discover potential therapeutic objectives; four mice were included in each group, and the specific model construction process and the administration grouping scheme are shown in Figure 4A. The arthritis score, arthritis swelling number and paw thickness were used to initially assess the role of HDAC6 shRNA in alleviating clinical disease in CIA mice (Figures 4B–E). Treatment was started on Day 30 after the first immunization, and the model group continued to develop arthritis, same as previous studies; the arthritis severity scores of CIA mice injected with HDAC6 shRNA were notably lower than those of the other groups. For the purpose of investigating the effect of HDAC6 shRNA treatment on the joints, collected ankle joint tissues from the right hind limb, and stained with H&E, toluidine blue and saffron-solid green. The ankle data showed (Figure 4F) that the degree of and inflammatory infiltration was lower in the ankle joints of the HDAC6 shRNA group than in those of the model group (*p* < 0.01). Toluidine blue and senna-solid green staining data demonstrated (Figure 4F) that the degree of bone damage and cartilage loss was also lower in the HDAC6 shRNA group (*p* < 0.01).

### HDAC6 loss inhibits the autophagy of molecular chaperones in the joint tissues of CIA mice

**FIGURE 5:**
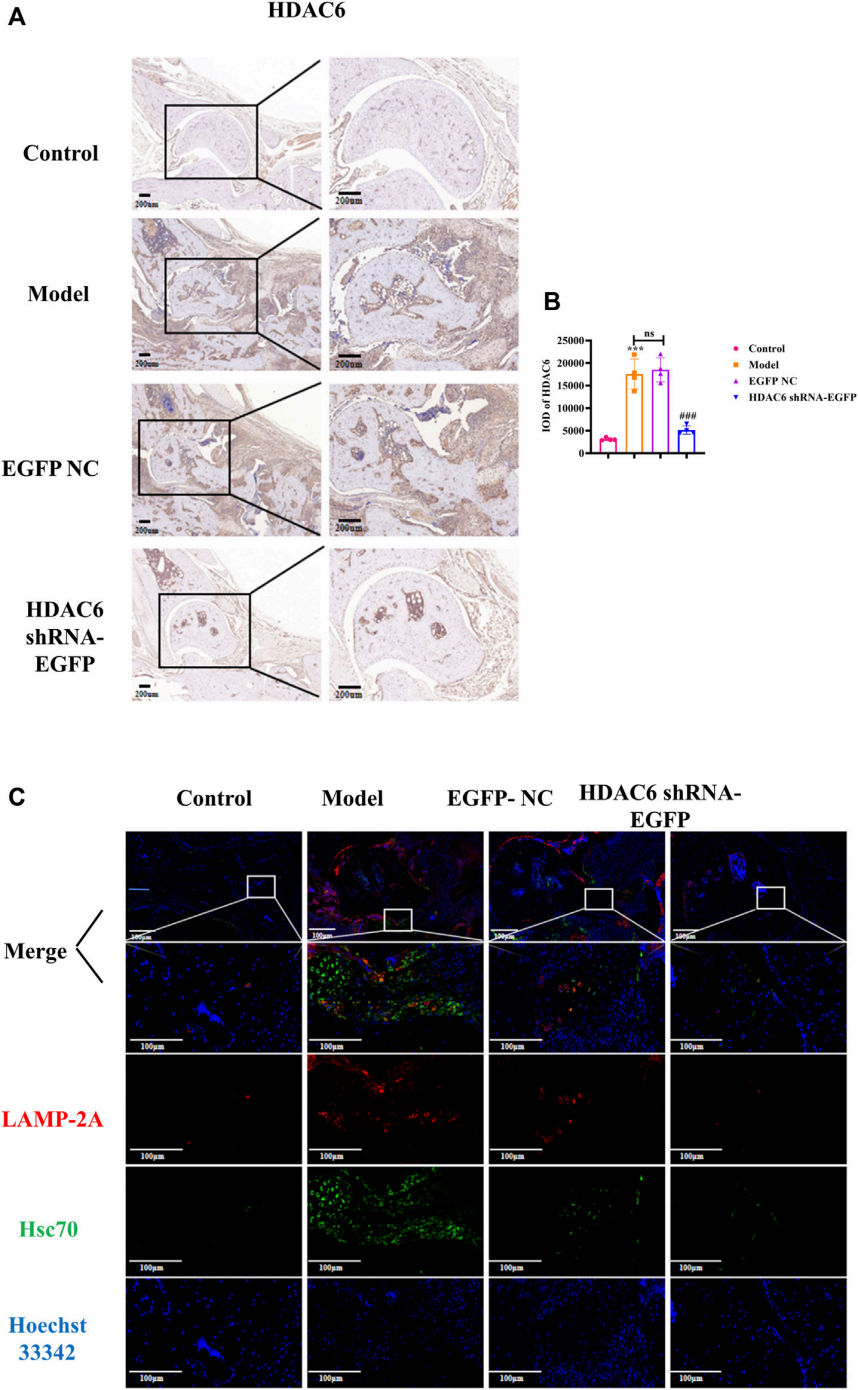
HDAC6 shRNA alleviates ankle synovitis and bone damage in CIA mice by regulating the expression of the molecular chaperone autophagy-associated proteins **(A)** Representative immunohistochemical staining of HDAC6 in synovial slices. **(B)** Statistical analysis of the quantified immunohistochemical staining for HDAC6 in synovial tissue. **(C)** On the 42nd day, representative LAMP-2A and Hsc70 hind paws were obtained from the HDAC6 shRNA group. ^***^ *p* < 0.001 vs. the control group; ^##^ *p* < 0.01, ^###^ *p* < 0.001 vs. the CIA group.

To investigate the effect of HDAC6 shRNA treatment on the joints, tissue from the ankle joints of the right hind limb of mice was subjected to immunohistochemical staining to detect HDAC6 protein expression (Figure 5A). The expression level of HDAC6 was upregulated in the synovium of the ankle joints of the model group compared with that in the control group; however, its expression was significantly reduced by the administration of HDAC6 shRNA (*p* < 0.001). As shown by ELISA (Figure 5 C), HDAC6 shRNA treatment effectively reduced the serum level of the HDAC6 factor, indicating that HDAC6 shRNA effectively inhibited the serum HDAC6 level in CIA mice (*p* < 0.05). To study the level of the molecular chaperone autophagy-related proteins in the joints induced by HDAC6 shRNA treatment, ankle joint tissues from the right hind limb of the mice in each group were also collected and stained for the Hsc70 and LAMP-2A proteins by immunofluorescence. Immunofluorescence staining of ankle joints from Hsc70 and LAMP-2A-treated mice was downregulated in the ankle joints of the HDAC6 shRNA group compared with those of the model group (Figure 5D). The outcomes indicated that HDAC6 shRNA could effectively downregulate the effect of the molecular chaperone autophagy in the joint tissues of CIA mice.

## Discussion

Previous studies have demonstrated that in the joints of RA patients, synovial tissues abnormally proliferate, 2/3 of the cells in the proliferated tissues are FLSs. For instance, TNF-α and IL-1β are known to induce the abnormal proliferation and migration of FLSs, as along with the production of various cytokines like IL-6 and TNF-α, thereby altering the synovial microenvironment (Suto et al., 2022). RA fibroblastic-like synoviocytes (RA-FLS) are highly specialized mesenchymal cells in the synovium of sensitive joints that are fully specialized mesenchymal cells with strong invasive ability and play an key part in the development of RA (Grzelecki et al., 2021). RA-FLS cell secrete a multitude of pathogenic mediators, such as cytokines (tumor necrosis factor alpha (TNF-α) and interleukin-6 (IL-6), which causes the migration and differentiation of other cells in the synovial membrane of RA patients, speeding up the progression RA (Leo et al., 2022). RA-FLS migrate and invade neighboring bone tissue, subsequently causing skeleton erosion and joint destruction. Consequently, inhibition of FLS-mediated proinflammatory response may be a novel therapeutic approach. The most damaged cells in RA are chondrocytes and synoviocytes (Ma et al., 2022).

HDAC6, the singular of the HDAC family situated in the cytoplasm, functions specifically to catalyze the deacetylation of nonhistone substrates (Zheng et al., 2023) and is involved in many physiological or pathological mechanisms in a wide range of diseases (Li et al., 2023a). Inhibitors of HDAC6, which exhibit immune modulatory effects in preclinical models, have a selective targeting effect without significant toxicity and prolong the stabilization of the disease in some patients (Tsimberidou et al., 2021). HDAC6 inhibitors have been indicated to inhibit matrix metalloproteinase activity, interrupt cytokine-induced signaling pathways, and mitigate cartilage degradation (Barter et al., 2022). Studies have demonstrated that inhibiting HDAC6 also improves the inflammatory environment triggered by IL-1β and modulates inflammatory signaling in chondrocytes (Zhang et al., 2023). Several reports have shown that the inhibition of HDAC6 with ustekin effectively alleviates cartilage damage (Shen et al., 2021). Therefore, inhibiting the expression of HDAC6 can effectively ameliorate synovial inflammation in rheumatoid arthritis patients.

In previous study, we found that RA-FLS are effective at studying RA *in vitro*; therefore, we utilized these cells as a model of rheumatoid arthritis. We used CAY10603, a highly efficient and specific inhibitor of HDAC6, to study the relationship between HDAC6 and synovial inflammation. RA-FLS play a vital function in the process of RA, and their overproliferation results in a large increase in cell number, which is the main cause of synovial tissue proliferation. We detected that 10 μM CAY10603 effectively attenuated the proliferative effect of RA-FLS. TNF-α and IL-6 play pivotal roles in inflammation and bone destruction in RA because they facilitate the release of subsequent inflammatory cytokines and inflammatory responses, contributing to tissue injury, which is closely associated with RA disease progression (Li et al., 2023b). We measured the supernatant of RA-FLS by ELISA and found that CAY10603 downregulated the secretion of TNF-α and IL-6 inflammatory factors in RA-FLS, further suggesting that CAY10603 could delay the onset of RA, which was consistent with the expected results. To better investigate the therapeutic effects of HDAC6 shRNA on RA, this chapter used a CIA mouse model with similar clinical signs and histopathological and immunological features as those of human RA (Choudhary et al., 2018). Present study showed that the mean arthritis score, number of swollen joints and paw thickness were significantly lower in HDAC6 shRNA-treated mice than in PBS-treated CIA mice. In addition, cytokines play important roles in RA, and we regarded cytokine levels as important indicators of RA. The serum levels of HDAC6 were significantly lower in the CIA mice in the HDAC6 shRNA group than in those in the CIA group. RA is characterized by chronic synovitis and progressive cartilage and bone erosion. Continued inflammation finally result in synovial hyperplasia, joint deformity and disability (Firestein and McInnes, 2017). Therefore, we used pathological staining to observe the effect of HDAC6 shRNA on arthritis in CIA mice. HE staining revealed thickening of the synovial membrane of the ankle joint, increased generation of vascular opacities, increased cellular infiltration, and a quantity of inflammatory cells in the vascular wall in the ankle joints of CIA mice compared with those in the normal group of mice. HDAC6 shRNA significantly inhibited inflammatory infiltration in the joints, synovial membrane hyperplasia, and vascular opacity formation. Moreover, toluidine blue staining and solid green staining revealed that HDAC6 shRNA could alleviate cartilage loss and bone destruction. In summary, these results indicate that the downregulation of HDAC6 can alleviate rheumatoid arthritis. By what mechanism does HDAC6 act? We further explored the mechanism of action of HDAC6.

Under pathological circumstances, autophagy may be associated with the ripening, survival and proliferation of wide range of immune and nonimmune cells (Gao et al., 2022), and its dysfunction has been related to RA, and plays a crucial part in the pathogenesis of RA (Zhao et al., 2021). Among them, the myosin-like BCL2 interacting protein (Beclin-1) is a regulatory and specific gene involved in autophagy and is a key factor in the initiation of autophagy. The key protein is microtubule-associated protein light chain 3B (LC3B), which controls the formation of the autophagic membrane and the convergence of autophagosomes and lysosomes, and the production of LC3B is a hallmark of autophagy that indirectly reflects the degree and level of autophagy. Ubiquitin-binding protein 62 (sequestosome 1, p62) can be degraded in autophagic lysosomes and can specifically recognize ubiquitinated proteins. Western blot assays revealed that CAY10603 could upregulate the level of LC3B and downregulate the level of p62 when it was used to treat RA-FLS.

Molecular chaperone-mediated autophagy can be involved in regulating the development of diverse diseases. LAMP-2A is a CMA receptor and limiting factor, whereas Hsc70 is a key carrier of CMA. The pathway relies mainly on the association of the Hsc70-substrate complex to the lysosomal membrane receptor LAMP-2A. It has been found that CMA dysfunction can affect the production of proinflammatory factors (Wu et al., 2023). Our experimental results showed that in an *in vitro* model, the results illustrated that CAY10603 inhibited the expression of HDAC6, Hsc70, and LAMP-2A in RA-FLS. Moreover, confocal microscopy revealed that Hsc70 and LAMP-2A could significantly downregulate the expression of Hsc70 and LAMP-2A in RA-FLS after treating CAY10603. Similarly, to observe whether HDAC6 shRNA regulates RA through CMA, immunofluorescence was used to confirm that HDAC6 shRNA could reduce the degree to which Hsc70 and LAMP-2A colocalize in ankle joints. Our findings suggest that the downregulation of HDAC6 can regulate the expression of Hsc70 and LAMP-2A in CMA, thereby alleviating RA and providing joint protection.

**FIGURE 6:**
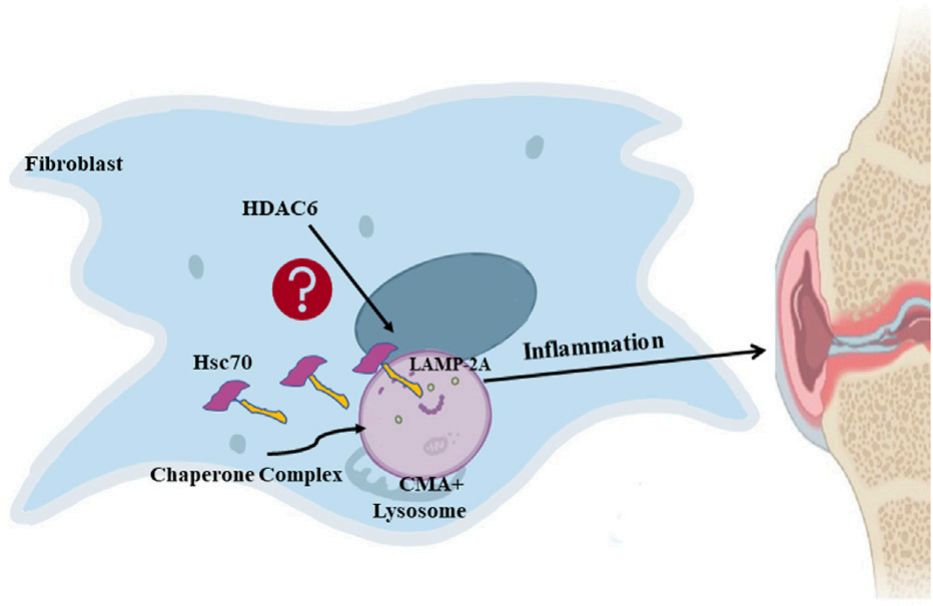
HDAC6 regulates rheumatoid arthritis through the CMA signaling pathway in RA-FLS and CIA mice.

Overall, we conclude that the inhibition of HDAC6 can downregulate CMA and thereby ameliorate rheumatoid arthritis HDAC6 may be a new potential target for the clinical treatment of rheumatoid arthritis. Figure 6.

## Data Availability

The original contributions presented in the study are included in the article/Supplementary materials, further inquiries can be directed to the corresponding authors.
